# Dupilumab for Atopic Dermatitis: The Silver Bullet We Have Been Searching for?

**DOI:** 10.7759/cureus.7565

**Published:** 2020-04-06

**Authors:** Ahsan Tameez Ud Din, Ifrah Malik, Daneyal Arshad, Asim Tameez Ud Din

**Affiliations:** 1 Internal Medicine, Rawalpindi Medical University, Rawalpindi, PAK

**Keywords:** atopic dermatitis, eczema, dupilumab, allergy

## Abstract

Atopic dermatitis is a chronic inflammatory condition of the skin affecting a large number of people worldwide. Historically, this condition has been managed by topical corticosteroids and general skincare measures. The inadequacy of these management strategies has always driven efforts to find better drugs. Dupilumab has been recently approved for the management of atopic dermatitis. It is a human monoclonal antibody that inhibits the binding of key interleukins involved in the pathogenesis of atopic dermatitis, thus blocking the signaling mechanisms and disrupting the disease progression. Dupilumab reduces the severity and associated symptoms of atopic dermatitis. It improves the life quality of patients and reduces the anxiety associated with the disease. Combination therapy of dupilumab and topical corticosteroids is more effective than dupilumab monotherapy. The treatment-related adverse events include headache, injection site reaction, conjunctivitis, nasopharyngitis, and herpes viral infections. Moreover, the simultaneous use of live vaccines with dupilumab is contraindicated. It is also beneficial in the management of asthma, chronic rhinosinusitis, and eosinophilic esophagitis. In this review, we have discussed the clinical efficacy and safety profile of dupilumab in the management of atopic dermatitis.

## Introduction and background

Atopic dermatitis is a chronic inflammatory condition of the skin characterized by pruritus and skin changes [1]. It affects 2-10% of adults worldwide and may be associated with other systemic disorders like asthma [2]. The underlying pathology of atopic dermatitis revolves mainly around type 2 immune-mediated reaction, with cytokines interleukin-4 (IL-4) and interleukin-13 (IL-13) playing a key role in its pathogenesis [3,4].

Owing to the chronic nature of the disease, the treatment of moderate-to-severe atopic dermatitis tends to be long term. Historically, topical corticosteroids and general skincare by emollients and non-irritative skin preparations have been the mainstay of management of atopic dermatitis [1,5,6]. The use of systemic agents (e.g., oral corticosteroids and immunosuppressants such as cyclosporine and methotrexate) and phototherapy are usually limited to the treatment of cases showing minimal response to the topical agents [7]. Long-term use of systemic agents is not recommended due to the risk of adverse side effects [5,7]. The inadequacy of the current mainstream treatment options has been the driving force behind the extensive efforts to find a safe and effective drug for the treatment of this ubiquitous skin disorder.

Dupilumab has been recently approved by the U.S. Food and Drug Administration (FDA) for the use in the treatment of atopic dermatitis. It is a human monoclonal antibody that targets the signaling mechanisms of cytokines IL-4 and IL-13 [8]. This review article aims to improve the understanding of this newly approved drug by comparing the findings of different clinical trials. The main objective of this study is to give a comprehensive view of the efficacy and safety profile of dupilumab.

## Review

Methods

A literature search was performed using MeSH (Medical Subject Headings) terms and keywords on the PubMed database. A total of 20 articles were identified. The selected studies included clinical trials that demonstrated the efficacy, side effects, drug interactions, and other uses of dupilumab.

Two independent reviewers screened the abstracts and the full text of articles. After meticulous screening and a detailed discussion, eight studies were selected for the final review.

One author performed data extraction, whereas a second author analyzed the extracted data. The study variables that were analyzed included the name(s) of the author(s), year of study, design of the study, sample size, the severity of atopic dermatitis, efficacy, adjunctive therapy, safety, and drug interactions.

Mechanism of action

Dupilumab is a human monoclonal antibody acting on the IL-4 receptor. Two types of IL-4 receptors present on a surface of many cells (e.g. B lymphocytes, eosinophils, dendritic cells, monocytes, macrophages, basophils, keratinocytes, etc.) are involved in the pathogenesis of type 2 allergic responses [9]. The binding of cytokine ligands to these receptors leads to a cascade of events that result in IgE (immunoglobulin E) class switching, TH2 (T-helper cell type 2) differentiation, and M2 macrophage polarization [10]. In atopic dermatitis, cytokines IL-4 and IL-13 play a key role in the modulation of the epidermal barrier and the stimulation of dermal inflammation and remodeling [11-13].

Evidence from a large number of clinical trials suggests that dupilumab acts by inhibiting the binding of IL-4 receptors to the main cytokines responsible for mediating the disease (IL-4 and IL-13). This results in the blockage of the signaling pathways, which disrupts the development and progression of allergic disorders [14].

Route of administration and recommended dose

Dupilumab is administered subcutaneously by injection. The patient may be taught the appropriate method of self-injection, which may reduce the inconvenience of repeated visits to a health care facility. The recommended dose of dupilumab is 300 mg every two weeks after an initial loading dose of 600 mg (two 300 mg injections at different sites) [15]. However, certain studies suggest that the dose of 300 mg given weekly may be as or even more effective than the current recommended dosing schedule [16,17].

Clinical efficacy and quality of life

The severity of atopic dermatitis and its effect on the quality of life is assessed by a variety of scoring systems that include subjective as well as objective measurements [18-22]. Table 1 lists the scoring systems frequently used by the investigators in the clinical trials concerning atopic dermatitis.

**Table 1:** Scoring systems used in atopic dermatitis trials EASI, Eczema Area and Severity Index; IGA, Investigator Global Assessment; SCORAD, Scoring Atopic Dermatitis; POEM, Patient-Oriented Eczema Measure; DLQI, Dermatology Life Quality Index; HADS, Hospital Anxiety and Depression Scale; EQ-5D-3L, 5-dimension 3-level EuroQol; NRS, Numeric Rating Scale

Scoring system	Description
EASI	Assesses both lesional intensity and extent of the disease. Four signs (erythema, papulation/edema, excoriation, and lichenification) are assessed and a score ranging from 0 to 3 is given [18,19].
IGA	Quick and simple “snapshots” of disease severity.
SCORAD	In addition to the signs assessed in the EASI, this scoring system also assesses oozing/crusting and xerosis of non-lesional skin [19].
POEM	Assesses the signs of crusting/weeping/exudation, lichenification, dryness, scaling, cracking/fissuring along with their effect on sleep.
DLQI	Consists of 10 queries that assess the patient’s perception of the effects of the specific skin condition on their health-related quality of life over the past week [20].
HADS	Checks for symptoms of anxiety and depression.
EQ-5D-3L	Consists of five dimensions of health status which include mobility, self-care, usual activities, pain/discomfort, and anxiety/depression [21].
Pruritus NRS	This is popular due to its simplicity. Patients are asked to rate the intensity of their itch using this scale from 0 (no itch) to 10 (worst imaginable itch) [22].

The clinical trials of dupilumab reported a reduction in the severity and improvement in the quality of life after using the drug. In a randomized, placebo-controlled, double-blind study by Thaçi et al., subjects were divided into six groups: five drug groups (having different dosing schedules) and one placebo group. The mean change in the Eczema Area and Severity Index (EASI) and Scoring Atopic Dermatitis (SCORAD) in the group receiving the dose of 300mg/week versus placebo at 16 weeks was found to be -55.7% and -43.1%, respectively. Quality of life was assessed by the Dermatology Life Quality Index (DLQI) score. A mean change of -61.6% as compared with the placebo was reported [23].

Beck et al. assessed the effects of dupilumab monotherapy as well as combination therapy (dupilumab with topical corticosteroids). The combination therapy was found to be most effective with 100% of patients achieving EASI-50 score (50% reduction in EASI score), which was double as compared with the patients receiving topical corticosteroids with placebo (p = 0.002). Pruritus scores reduced by 55.7% in the dupilumab group versus 15.1% in the placebo group (p < 0.001) [24].

A phase 3 trial conducted by Blauvelt et al. for one year reported that patients receiving dupilumab with topical corticosteroids achieved co-primary endpoints of Investigator Global Assessment (IGA) 0/1 and EASI-75 (75% reduction in EASI score) in a significantly higher number than the placebo groups (p < 0·0001). Quality of life improved by 10.5 on DLQI score in the group receiving 300 mg weekly dose of the combination therapy (dupilumab plus topical corticosteroids) [25].

Guttman-Yassky et al. assessed skin biopsy specimens and blood samples from 54 patients who were randomly divided into the dupilumab and placebo groups. At 16 weeks, the lesional epidermal thickness was found to be significantly reduced in the dupilumab group compared with the placebo group (p = 0.0002). The atopic dermatitis severity scores on EASI and SCORAD were also found to be notably reduced after the use of dupilumab [26].

Hamilton et al. performed transcriptome analyses of skin biopsy specimens from patients before and after treatment with dupilumab or placebo. It was found out that the expression of the genes normally involved in atopic dermatitis was reduced by 65% in the patients using 300 mg weekly dose of dupilumab. The changes in clinical scores were also indicative of a reduction in disease severity upon using dupilumab. A change of -64.3% was observed in the EASI score assessed after four weeks in the group using 300 mg of dupilumab weekly [27].

In a double-blind, parallel-group dose-ranging study conducted in 2016 by Simpson et al., 380 patients were divided into six different groups (five drug groups with one placebo group). Patients using 300 mg dupilumab weekly reported the most improvement in the symptoms of atopic dermatitis and the quality of life of patients. The Pruritus NRS (Numeric Rating Scale) score was significantly reduced (p < 0.0001) in most of the drug groups. There was a reduction of 75% in the anxiety score on Hospital Anxiety and Depression Scale (HADS) in patients using 300 mg dupilumab weekly as compared with 22.2% improvement seen in the placebo group (p < 0.05). The quality of life was also found to be significantly improved as evident from the DLQI and 5-dimension 3-level EuroQol (EQ-5D-3) scores (p < 0.05) [28].

Table 2 summarizes the efficacy of dupilumab in terms of the commonly used scoring systems as reported in the studies mentioned. The table includes the results reported for the weekly dose of 300 mg dupilumab with the exception of Guttman-Yassky et al. (200 mg weekly).

**Table 2:** Efficacy of dupilumab Combination therapy refers to dupilumab plus topical corticosteroids. SCORAD, Scoring Atopic Dermatitis; EASI, Eczema Area and Severity Index; Pruritus NRS, Pruritus Numeric Rating Scale; DLQI, Dermatology Life Quality Index; IGA, Investigator Global Assessment; EASI-50, 50% reduction in EASI score; EASI-75, 75% reduction in EASI score; SCORAD sleep VAS, Scoring Atopic Dermatitis sleep Visual Analogue Scale

Authors	Year	Efficacy				
		Change in SCORAD (from baseline)	Change in EASI score	Change in Pruritus NRS score (from baseline)	Change in DLQI score (from baseline)	Patients achieving IGA score 0 or 1
Thaçi et al. [23]	2016	−56·9%	–73.7% (from Baseline)	−46·90%	−59·0%	33%
Beck et al. [24]	2014		Combination therapy EASI-50 = 100% (p < 0.05) Dupilumab monotherapy EASI-50 = 85% (p < 0.001) Combination therapy EASI-75 = 62% Dupilumab monotherapy EASI-75 = 62%	Dupilumab monotherapy = −55.7% Combination therapy = −70.7% (p < 0.05)		Dupilumab monotherapy = 40% (p < 0.001) Combination therapy = 52%
Blauvelt et al. [25]	2017	Combination therapy = −63·3% (p < 0·0001)	Combination therapy EASI-75 = 64% (p < 0·0001) and EASI-50 = 78% (p < 0·0001)	Combination therapy = −4·1 (p < 0·0001)	Combination therapy = –10.5 (p < 0.0001)	Combination therapy = 39% (p < 0·0001)
Guttman-Yassky et al. [26]	2019	−54.8% (p < 0.0001)	EASI-75 = 66.7% (p = 0·0001) and EASI-50 = 77.8% (p < 0·0001)	−51.5% (p = 0.0027)		37.0% (p = 0.0006)
Davis et al. [29]	2018		EASI-75 = 78.6% and EASI-50 = 92.9%			50%
Hamilton et al. [27]	2014		EASI-50 = 71.4%			
Simpson et al. [28]	2016	SCORAD sleep VAS = –4.3 (p < 0.0001)		–3.2 (p < 0.0001)	–9.3 (p < 0.0001)	
Blauvelt et al. [30]	2019		EASI-50 = 72.2% and EASI-75 = 53.6%	–4.46		44.30%

Safety and drug interactions

Evidence from several trials suggests that treatment with dupilumab results in certain treatment-emergent adverse events. The most commonly reported adverse effects of dupilumab include headache, injection site reaction, conjunctivitis, and nasopharyngitis [23-26]. Herpes viral infections have also been reported by some researchers [23,26].

Due to the potential effects of dupilumab on the cytochrome P450 (CYP450) enzyme system, FDA recommends close monitoring of patients taking concomitant CYP450 substrate drugs (such as warfarin and cyclosporine) [15]. In contrast, a study conducted by Davis et al. in 2018 failed to demonstrate any meaningful effect of dupilumab on the pharmacokinetics of CYP450 substrates [29].

A study by Blauvelt et al. concluded that dupilumab did not affect the responses to the non-live vaccines included in the trial [30]. The current recommendations by FDA caution only against the use of live vaccines in patients taking dupilumab [15].

Other uses

An increasing number of clinical trials suggest that the use of dupilumab reduces the severity of various allergic conditions due to its targeted action on specific cytokines. Corren et al. reported a significant improvement in the severity of asthma and the quality of life of the patients [31]. Other trials have similarly assessed the efficacy and safety of dupilumab in patients with uncontrolled asthma [32,33]. Similarly, the beneficial effects of dupilumab were reported in patients with conditions such as chronic rhinosinusitis and eosinophilic esophagitis [34,35].

## Conclusions

Dupilumab is a recently approved drug for the management of atopic dermatitis, which works by inhibiting the signaling mechanisms involved in the pathogenesis of allergic skin conditions. The evidence from an increasing number of trials suggests that the benefits of dupilumab far outweigh its side-effects. It may be too early to declare dupilumab the ultimate drug of choice for atopic dermatitis, but the current evidence suggests that it has the potential to be the next wonder drug in dermatology.
